# Utilization of a CRISPRi-based *ex vivo* challenge model to reveal temporally dependent gene essentiality in intracellular *Mycobacterium tuberculosis*

**DOI:** 10.1128/mbio.00610-26

**Published:** 2026-04-20

**Authors:** Monique E. Theriault, Andrew I. Wong, Michael A. DeJesus, Davide Pisu, Bom Nae Rin Lee, Greana Kirukubar, Shuqi Li, Joshua B. Wallach, Dirk Schnappinger, Gabrielle Lê-Bury, David G. Russell, Jeremy M. Rock

**Affiliations:** 1 Department of Microbiology and Immunology, College of Veterinary Medicine, Cornell University 728647, Ithaca, New York, USA; 2 Laboratory of Host-Pathogen Biology, The Rockefeller University 5929, New York, New York, USA; 3 Department of Microbiology and Immunology, Weill Cornell Medicine 12295, New York, New York, USA; Universiteit Gent, Gent, Belgium

**Keywords:** CRISPR genetic screen, *Mycobacterium tuberculosis*, macrophage, granuloma

## Abstract

*Mycobacterium tuberculosis* (Mtb) remains a leading cause of infectious disease mortality worldwide, largely due to its ability to survive within host macrophages. Despite advances in understanding the environmental pressures Mtb encounters *in vivo*, the genetic requirements for adaptation and survival within the intracellular niche remain incompletely defined. Here, we employed a genome-wide CRISPR interference (CRISPRi) screen in an *ex vivo* model exploiting single-cell suspensions from Mtb-infected mouse lung homogenates to identify genes critical for intracellular survival at different time points in the infection continuum. Using a library comprising ~20,000 sgRNAs covering >96% of Mtb open reading frames, we identified genes required for growth within the changing immune microenvironment. Mutant depletion patterns varied across immune environments sampled at 2, 4, and 6 weeks post-infection, which revealed a weighted dependency on cell wall biosynthesis genes early and the reliance on cholesterol catabolism and iron acquisition across all time points. Functional validation of three genes—*embB*, *fadE29*, and *mbtI*—confirmed their temporal significance *in vivo*. This screen provides increased resolution of the differential metabolic vulnerabilities in Mtb in the evolving immune environments during infection, stressing the temporal nature of conditional essentiality *in vivo*.

**IMPORTANCE:**

*Mycobacterium tuberculosis* (Mtb) remains a leading cause of infectious disease mortality worldwide, largely due to its ability to survive within host macrophages. Despite advances in understanding the environmental pressures Mtb encounters *in vivo*, the genetic requirements for adaptation and survival within the intracellular niche remain incompletely defined. Here, we employed a genome-wide CRISPR interference (CRISPRi) screen in an *ex vivo* model exploiting single-cell suspensions from Mtb-infected mouse lung homogenates to identify genes critical for intracellular survival at different time points in the infection continuum. This novel approach enabled us to identify how different bacterial metabolic pathways were of greater importance to the bacterium at different time points post-infection. The results provide insights into how the evolving immune response to infection shapes the metabolic and replicative status of the bacterium. This information has significance in the design of therapeutic strategies toward cure.

## INTRODUCTION

*Mycobacterium tuberculosis* (Mtb), the etiologic agent of tuberculosis (TB), remains a leading global health threat, with over 10 million new cases and 1.3 million deaths annually (1). Its remarkable success as a human pathogen stems from its ability to persist within its host without necessarily progressing to active disease (2, 3). This persistence is facilitated by a broad range of adaptive mechanisms that allow Mtb to endure nutrient limitation, oxidative stress, hypoxia, and immune pressures encountered within the host environment (4, 5).

A comprehensive understanding of the genetic factors required for Mtb’s intracellular survival is important for developing new therapeutic strategies, particularly given the rise of multidrug-resistant TB. Following from the pioneering study of Sassetti and Rubin (6), several labs have exploited Tn-seq screens of Mtb infection *in vivo* designed to interrogate different aspects of the host–pathogen interplay (7–10). However, in each instance, the screen design has had to accommodate the challenges associated with the non-physiological nature of the infection conditions required to achieve sufficient coverage, even with non-genome-wide libraries. Given the diverse nature of the design of these screens, we felt there were still scientific questions to be addressed through genetic screening of *in vivo* infection environments. Moreover, the current screen is designed to reveal bacterial loci preferentially required at differing immune states reflecting the evolving immune response to infection. We believe that this has relevance for infection in individuals previously exposed to or infected by Mtb, which likely represents the majority in high-transmission settings such as sub-Saharan Africa (11, 12).

CRISPR interference (CRISPRi) has emerged as a powerful and inducible tool for loss-of-function screening in Mtb (13, 14), and we felt it was particularly suitable for revisiting the bacterial response to the changing immune environments experienced by Mtb during progression of disease. One of the major challenges in performing *in vivo* CRISPRi or Tn-seq screens is the severe bottleneck in bacterial population diversity imposed by aerosol or intranasal infection routes, which typically deliver only 100–3,000 CFU to the lungs. This bottleneck limits genome-wide screening resolution and restricts coverage of large libraries. To circumvent this, we employed an immunologically informed *ex vivo* infection model—the deconstructed granuloma (DeGr) platform (15)—which uses lung-derived immune cell suspensions from Mtb-infected mice. This system retains key features of the *in vivo* tissue microenvironment, including macrophage diversity and inflammatory signaling, while permitting sufficient bacterial input for deep library coverage and robust selection analysis.

In this study, we performed a genome-wide CRISPRi screen using a high-complexity sgRNA library targeting over 3,900 Mtb genes, including coding and non-coding RNAs. We challenged the pooled library with DeGr lung suspensions isolated at 2, 4, and 6 weeks post-infection, capturing temporal variation in immune pressure during infection. Analysis of sgRNA depletion across time points revealed distinct classes of essential genes, including those involved in iron acquisition, cholesterol catabolism, and cell wall synthesis. To validate our findings, we constructed individual CRISPRi knockdown strains of *embB*, *mbtI*, and *fadE29* and demonstrated that repression of these genes attenuated bacterial growth *in vivo* in naïve and vaccinated mice. Our findings provide a systems-level view of Mtb genetic dependencies across the course of infection and highlight the value of CRISPRi for resolving conditional essentiality within complex immune environments, and the temporal changes in phenotype underscore the critical role of metabolic adaptation in Mtb pathogenesis.

## RESULTS

### A CRISPRi platform enables genome-wide functional screening in host-like environments

**Fig 1:**
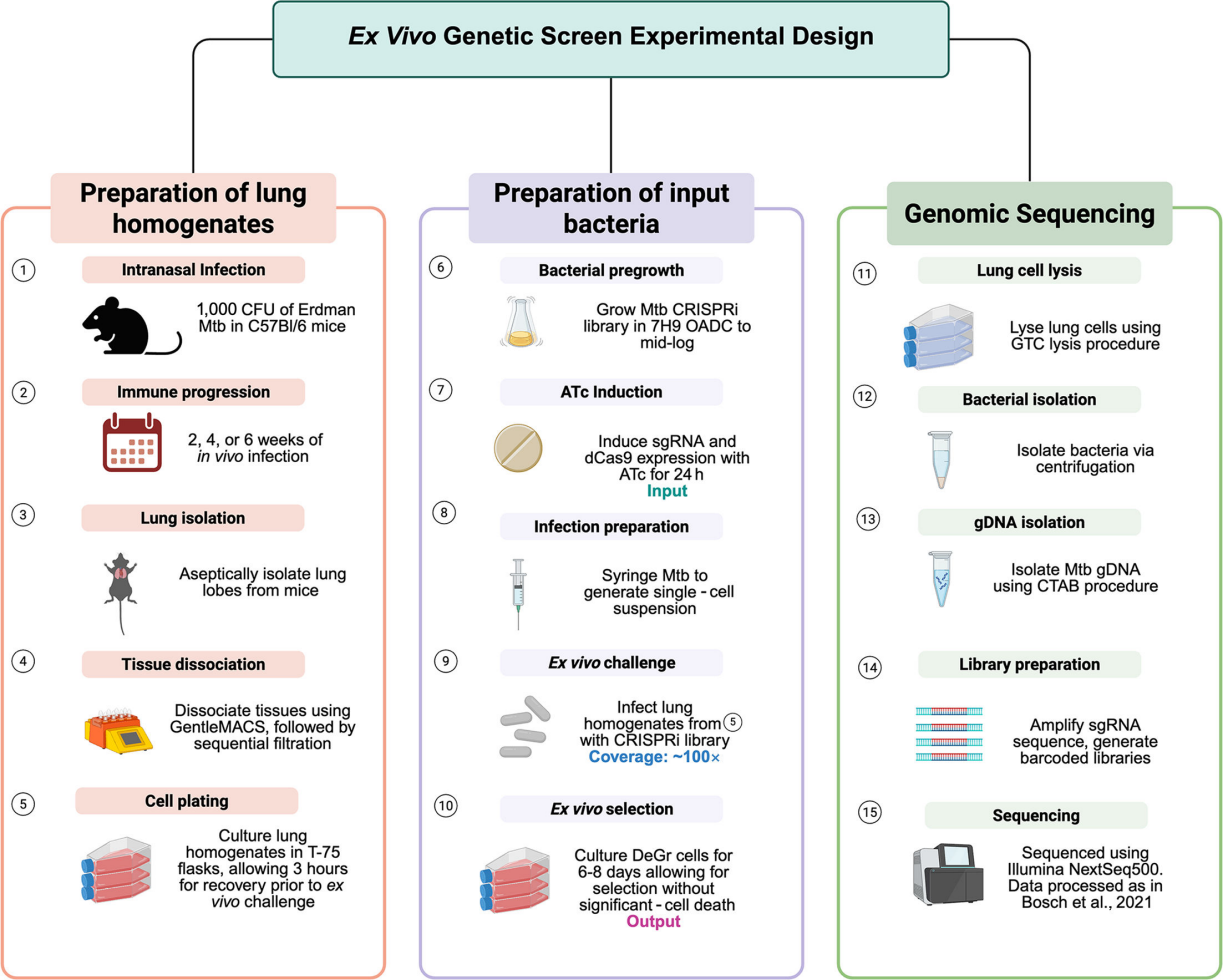
*Ex vivo* genetic screen experimental setup.

**Fig 2:**
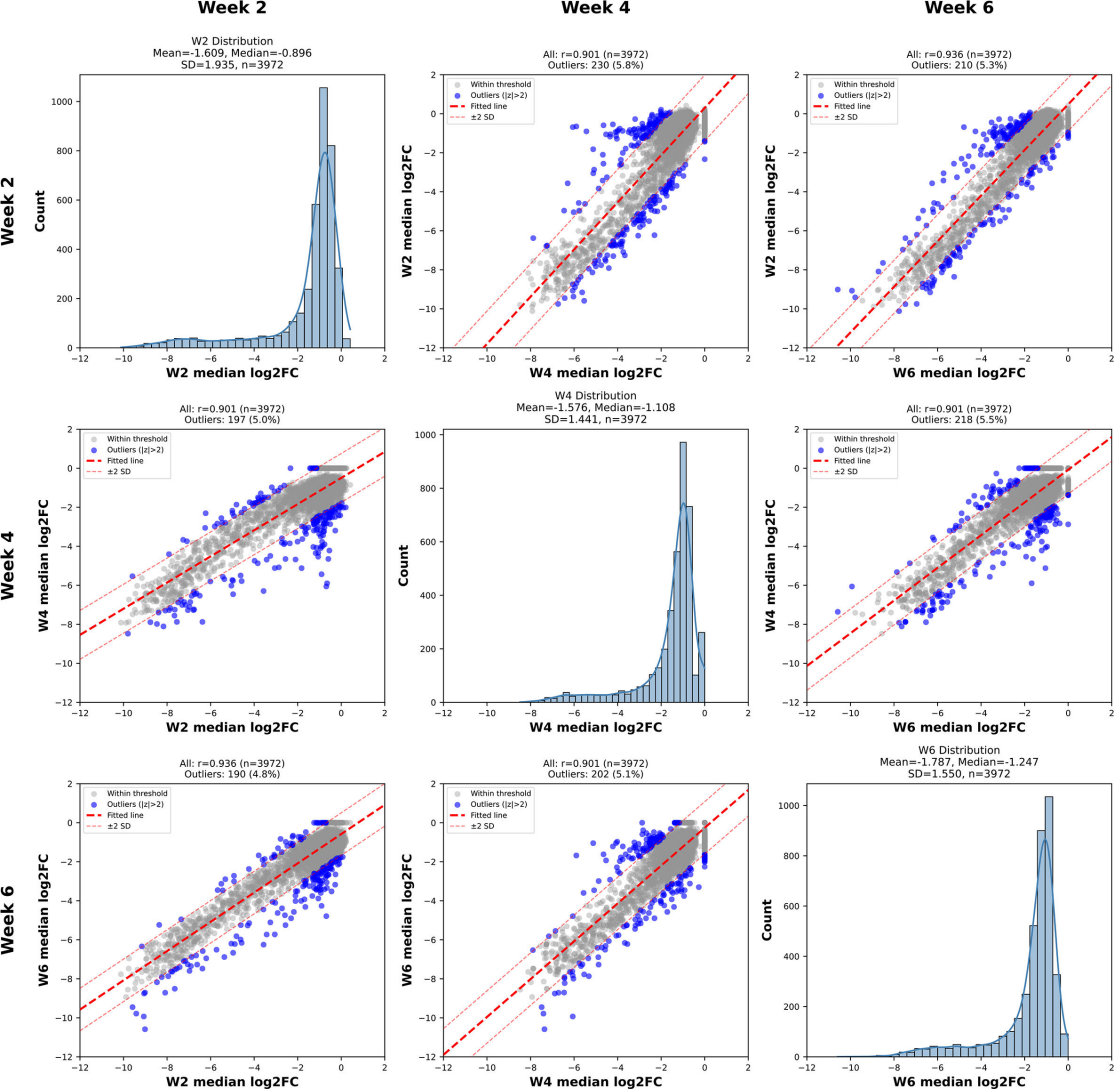
Reproducibility and correlation of genome-wide CRISPRi screens across infection time points. Median log_2_ fold change (log_2_FC) comparison grid showing correlations between replicate CRISPRi screens performed on *M. tuberculosis* in deconstructed granuloma (DeGr) cell suspensions at 2, 4, and 6 weeks post-infection. Scatterplots depict linear fits (dashed red lines) with outlier genes (>2 SD from mean) shown in blue. Histograms display overall log_2_FC distributions per time point (W2, W4, W6). High Pearson *r* values (≥0.9) confirm strong reproducibility and consistency of depletion patterns across technical and biological replicates.

To identify *M. tuberculosis* genes required for survival in the host, we performed a pooled CRISPR interference (CRISPRi) screen in an *ex vivo* infection model that recapitulates key aspects of the *in vivo* immune environment (15). This “deconstructed granuloma” (DeGr) system applied here used single-cell suspensions from intranasally infected mice at 2, 4, or 6 weeks post-infection (w.p.i.). This has been shown to be an extremely inclusive platform containing a full complement of innate and acquired immune cells (15), with the host macrophages changing in phenotype as a function of both origin and time post-infection (16–19). The time points of 2, 4, and 6 weeks were selected to capture the temporal shift from innate to adaptive immunity (17, 18). Using a library of ~20,000 sgRNAs targeting >96% of annotated genes with up to five sgRNAs per gene, we screened for gene knockdowns that exhibited reduced bacterial fitness following *ex vivo* infection of lung cell suspensions generated at the different time points post Mtb challenge (Fig. 1). Pairwise correlation analysis of biological and technical replicates demonstrated high reproducibility across all infection time points, with Pearson correlation coefficients ≥0.9 indicating consistent sgRNA depletion patterns and minimal batch variability between independent CRISPRi screens (Fig. 2).

**Fig 3:**
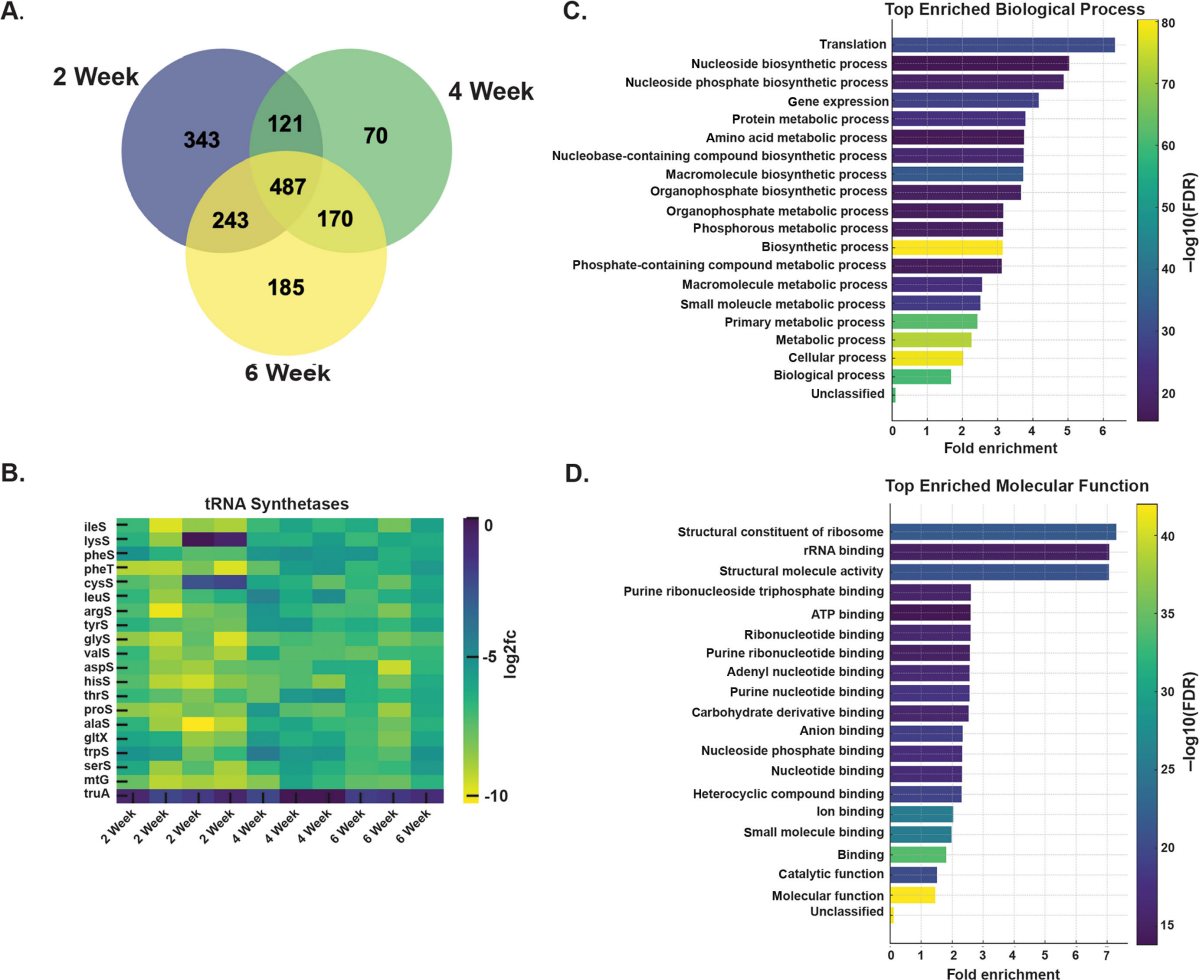
Identification of conserved and functionally enriched essential genes across infection time points. (**A**) Venn diagram showing overlap of *ex vivo* essential genes identified at 2, 4, and 6 weeks post-infection. Gene had to be depleted in at least two samples to be included. A substantial core of 487 genes was shared across all time points, while additional genes were uniquely essential at specific stages. (**B**) Heat map of log_2_ fold change (log2FC) values for *M. tuberculosis* tRNA synthetases, showing sustained *ex vivo* depletion across all time points. Each row represents a tRNA synthetase gene, and each column corresponds to a replicate at a given time point. Further details on how replicates were generated can be found in the Materials and Methods. (**C**) Gene ontology (GO) biological process enrichment among *in vivo* essential genes identified by PANTHER overrepresentation test. The top 20 most significantly enriched processes (FDR < 0.05) are shown, with color intensity reflecting statistical significance (−log_10_ FDR). (**D**) GO molecular function enrichment of the same gene set, highlighting functions involved in translation, nucleotide binding, and ribosomal structure. Fold enrichment and statistical significance are shown in panel C.

Analysis with MAGeCK revealed 487 genes consistently depleted across time points (Fig. 3A). These included central metabolic functions, translation machinery, and stress response genes (Fig. 3A; File S1) (5, 20). Pathway enrichment analysis highlighted ATP-dependent biosynthetic processes, tRNA synthetases, and cofactor biosynthesis (Fig. 3B through D), corroborating vulnerabilities described in nutrient-limited or host-like conditions (21, 22).

### Early intracellular infection selects for cell wall synthesis genes

**Fig 4:**
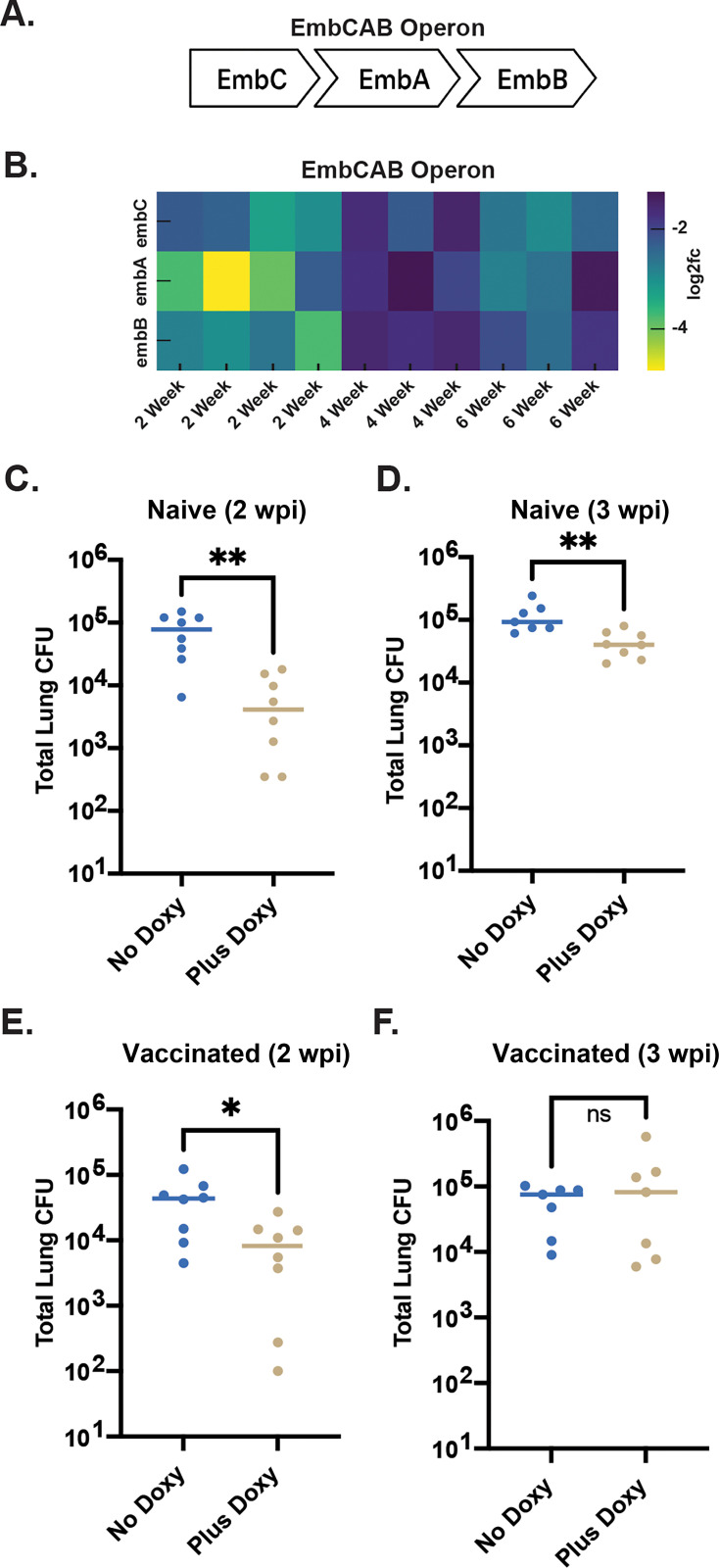
CRISPRi-mediated repression of *embCAB* genes impairs *M. tuberculosis* growth during establishment of infection. (**A**) Schematic of the *embCAB* operon in *M. tuberculosis*, encoding arabinosyltransferases EmbC, EmbA, and EmbB involved in cell wall biogenesis. (**B**) Heatmap showing log_2_ fold change in *sgRNA* abundance targeting *embC*, *embA*, and *embB* from 2, 4, and 6 weeks post-infection (w.p.i.) lung homogenates. (**C–F**) Total lung CFU in mice infected with CRISPRi *M. tuberculosis* strains targeting the *embCAB* operon. Mice were either untreated (No Doxy) or administered doxycycline (Plus Doxy) to induce *dCAS9* and *sgRNA* expression during infection. (**C**) Naïve mice at 2 w.p.i. (**D**) Naïve mice at 3 w.p.i. (**E**) Vaccinated mice at 2 w.p.i. (**F**) Vaccinated mice at 3 w.p.i. Each point represents a single mouse; bars show median values. Statistical significance determined by unpaired *t*-test. *P < 0.04, **P < 0.01; ns, not significant.

Although certain genes were classified as “unique” hits at a given time point, most of these genes still showed depletion at the other time points. Their effects simply did not cross the statistical threshold or cutoffs needed to be called hits at those stages. This is reflected in the strong diagonal structure of the scatterplots (Fig. 2), indicating that gene-level fitness effects were broadly consistent across time but showed stage-specific differences in depletion magnitude. The strongest stage-specific selection, which resulted in depletion of 343 knockdowns, was observed at the 2 w.p.i. time point, corresponding to the early innate phase of infection (Fig. 3A; File S1). This was unanticipated and, initially, appeared counterintuitive because one always imagines this early period of infection to be more permissive to Mtb survival. However, as shown in the replicate correlation grid (Fig. 2), the W2 histogram displayed a deeper left tail—indicating a greater maximum depletion range at week 2 compared to the 4 and 6 week time points—thus confirming the stronger selection and broader gene essentiality early in infection. Upon analysis, we noted that the *embCAB* operon, which encodes arabinosyltransferases involved in arabinogalactan biosynthesis, demonstrated markedly higher depletion at this stage compared to 4 and 6 week time points (Fig. 4B; File S3). Consistent with this hypothesis, previous Dual RNA-seq transcriptomic analysis reported elevated cell wall synthesis activity early in infection (17), and bacterial replication reporters demonstrate higher rates of division in early infection (16, 23). In combination, these data imply that this bacterial population is in a more replicative state, and furthermore, that survival in a replicative state is dependent on a larger proportion of the bacterial genome than survival under the selection pressure of a more developed immune response that is known to restrict bacterial replication (16).

To test this hypothesis experimentally, we generated a CRISPRi knockdown of *embB* and quantified the relative survival of induced and non-induced Mtb knockdowns in mouse infections over time and under differing immune pressure. Rather than use different time points of infection, we infected naïve versus vaccinated mice as an alternative route to test whether the differential phenotypes observed were a consequence of an acquired immune response. Consistent with our hypothesis, we observed a 1.5 log-fold drop in lung CFU at 2 w.p.i. in naïve mice, with reduced impact at 3 w.p.i. (Fig. 4C and D). In vaccinated animals, under selection of a pre-existing immune response, *embB* repression also impaired bacterial growth at 2 w.p.i. but not at 3 w.p.i. (Fig. 4E and F). These results emphasize the temporal nature of gene essentiality *in vivo* and the utility of time-resolved screening facilitated by the development of this current *ex vivo* challenge platform.

### Cholesterol catabolism is essential throughout intracellular infection

**Fig 5:**
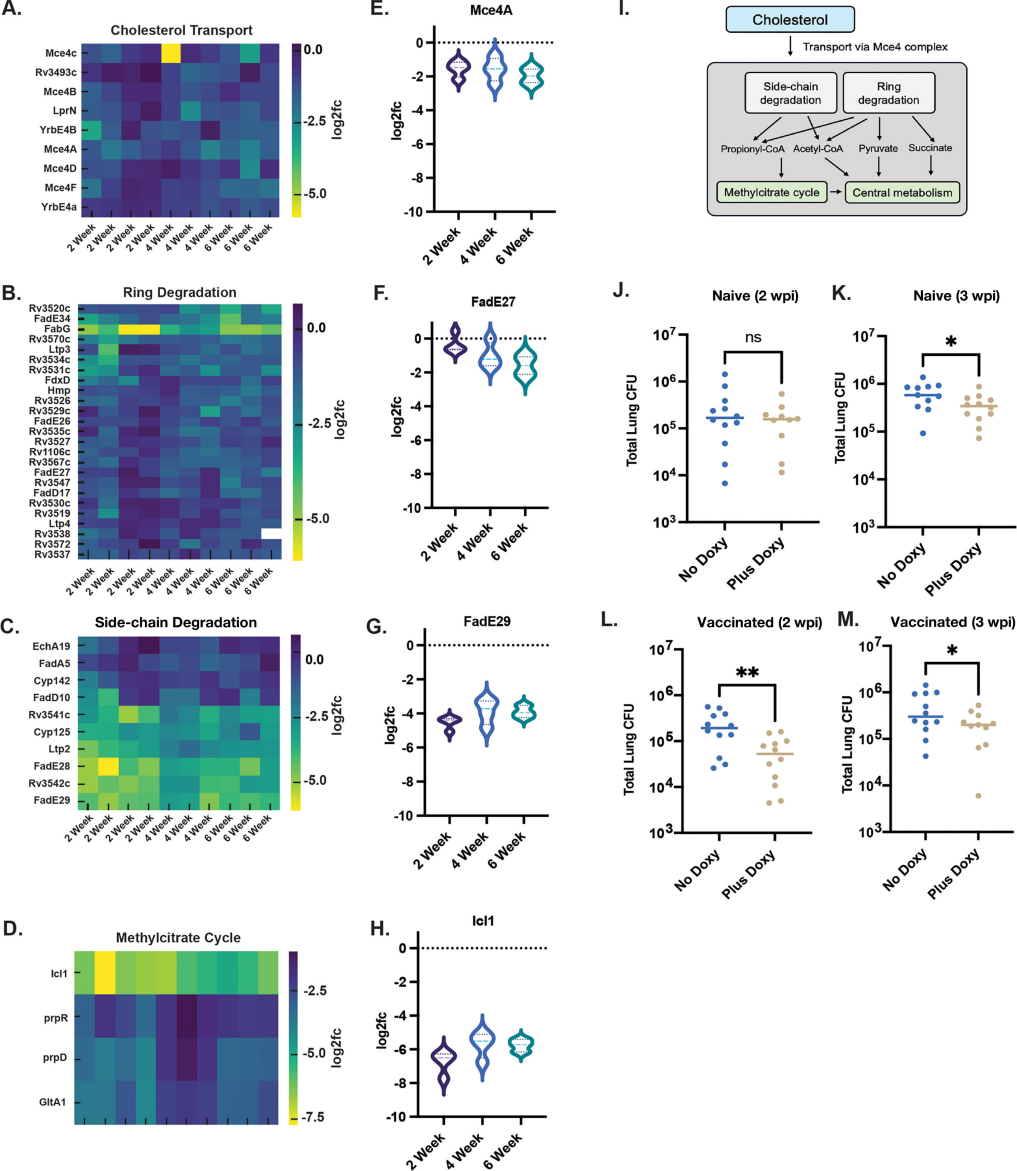
Cholesterol catabolism is a critical metabolic vulnerability for *M. tuberculosis* during intracellular infection. (**A–D**) Heatmaps showing log_2_ fold change in sgRNA abundance across 2-, 4-, and 6-week time points for genes involved in the four key pathways in the cholesterol catabolic process: transport (**A**), ring degradation (**B**), side-chain degradation (**C**), and the methylcitrate cycle (**D**). sgRNA abundance for representative genes from these pathways is displayed as violin plots for mce4A (**E**), fadE27 (**F**), fadE29 (**G**), and icl1 (**H**). (**I**) Schematic overview of the cholesterol catabolic process. (**J–M**) *In vivo* validation of *fadE29* gene essentiality. *FadE29* knockdown significantly reduced lung CFU in vaccinated mice at both 2 and 3 weeks post-infection and in naive mice at 3 weeks post-infection (**K–M**) but not in naive mice at 2 weeks post-infection (**J**) Statistical significance was assessed using unpaired *t*-tests; *P < 0.05, **P < 0.01; ns, not significant.

Mtb relies heavily on host-derived lipids during infection, and cholesterol, in particular, is critical for survival within macrophages (24, 25). Our screen revealed consistent depletion of genes involved in cholesterol uptake and catabolism across all time points (Fig. 5A through H), including *fadE28*, *fadE29*, and genes in the methylcitrate cycle such as *prpC* and *icl1*. These findings are in agreement with earlier work showing that the methylcitrate cycle is essential for detoxifying propionyl-CoA generated during cholesterol degradation (26–28). This also provides additional justification for the continued development of inhibitors of bacterial cholesterol metabolism as viable chemotherapeutic agents against Mtb *in vivo* (29).

Interestingly, we observed stronger depletion of cholesterol side-chain degradation genes than those involved in A/B ring cleavage (Fig. 5B/C and F/G), suggesting, in agreement with previous observations (30), that side-chain metabolism may represent a more stringent bottleneck *in vivo*. We constructed a *fadE29* knockdown strain and found that it had no significant impact in naïve mice at 2 w.p.i. but significantly impaired maintenance of the bacterial population at 3 w.p.i. and in vaccinated animals (Fig. 5J through M). This is consistent with dual-RNA-seq data that indicated that cholesterol breakdown supported bacterial survival under all conditions, while fatty acid catabolism fueled the establishment of infection and its active growth (5, 18).

### Iron acquisition via mycobactin synthesis and transport is required *in vivo*

**Fig 6:**
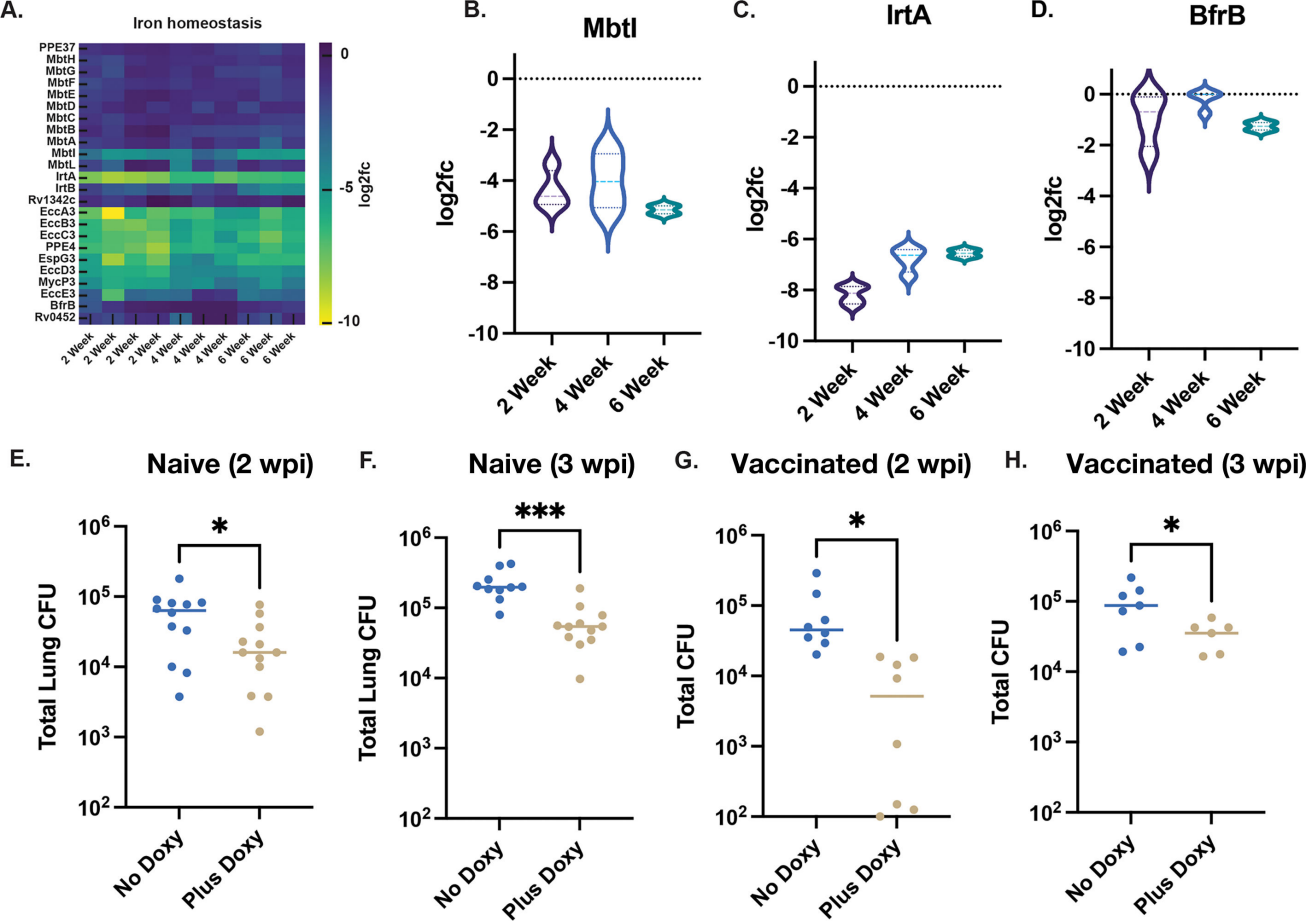
Iron acquisition genes are consistently depleted *in vivo,* and *mbtI* knockdown impairs *M. tuberculosis* fitness in mice. (**A**) Heatmap showing log_2_ fold change values of sgRNA abundance for genes involved in iron homeostasis across all biological replicates and time points (2, 4, and 6 weeks post-infection). (**B–D**) Violin plots showing log_2_fc of sgRNAs targeting *mbtI* for mycobactin synthesis (**B**), *irtA* for iron import (**C**), and *bfrB* for iron storage (**D**). (**E–H**) Lung CFUs following CRISPRi knockdown of *mbtI* in *M. tuberculosis* infected mice. Mice were either untreated (No Doxy) or administered doxycycline (Plus Doxy) to induce *dCas9* and *sgRNA* expression during infection. (**E**) Naïve mice at 2 w.p.i. (**F**) Naïve mice at 3 w.p.i. (**G**) Vaccinated mice at 2 w.p.i. (**H**) Vaccinated mice at 4 w.p.i. Each point represents a single mouse; bars show median values. Statistical significance was determined by unpaired *t*-test. *P < 0.05, **P < 0.01; ns, not significant.

Intracellular *M. tuberculosis* experiences dynamic iron limitation, and bacterial iron acquisition pathways are key to virulence (31–33). We observed strong depletion of *irtA*, *irtB*, and ESX-3 genes (*eccA3–eccG3*) across all time points (Fig. 6A through D), in agreement with prior studies showing that disruption of ESX-3 impairs siderophore-mediated iron uptake (34, 35).

Among the mycobactin biosynthesis genes, only *mbtI*—encoding a salicylate synthase—was negatively selected against across all time points (Fig. 6B). This aligns with earlier biochemical data suggesting that *mbtI* catalyzes the rate-limiting step in siderophore synthesis (36, 37), and with Dual RNA-seq studies indicating that iron metabolism is tightly correlated with bacterial fitness *in vivo* (17). CRISPRi knockdown of *mbtI* significantly reduced lung CFU in both naïve and vaccinated mice (Fig. 6E through H), confirming its essentiality and supporting its role as a metabolic chokepoint under host-imposed iron limitation.

We noted only modest depletion of *bfrB*, which encodes a ferritin-like iron storage protein, at 2 and 6 weeks post-infection, but not at 4 weeks (Fig. 6D). This limited and stage-specific depletion suggests that, while *bfrB* may contribute to buffering transient iron availability or protecting against the Fenton reaction under oxidative stress at certain phases of infection, it is not universally essential across immune states. In contrast to the consistent depletion observed for *irtAB* and *mbtI*, the temporally variable depletion of *bfrB* indicates that iron storage is less critical than iron import for *M. tuberculosis* survival *in vivo*. These findings reinforce the notion that nutrient acquisition pathways, rather than nutrient storage systems, are the dominant determinants of fitness in the iron-limited host environment (38).

### Validation of metabolic vulnerabilities in distinct immune contexts

Together, our findings validate three metabolic genes with different essentiality profiles *in vivo*—*embB*, *fadE29*, and *mbtI* (Fig. 4C through F; Fig. 5J through M; Fig. 6E through H). Each knockdown impaired bacterial growth, but with different kinetics and immune context dependencies. These data further elucidate how the metabolic landscape of Mtb is shaped during the acquisition of an acquired immune response, as implied by recent single-cell and spatial transcriptomics studies (16–18). The DeGr platform, reliant on lung cell suspensions isolated at differing times post-infection, coupled with the inducible CRISPRi knockdown system, provides a powerful new strategy for determining gene essentiality as a dynamic, context-dependent spectrum rather than a static binary trait.

## DISCUSSION

The ability of *Mycobacterium tuberculosis* (Mtb) to adapt to and persist within the hostile intracellular environment of host macrophages lies at the core of its remarkable success as a human pathogen. However, our appreciation of those specific bacterial genes required for *in vivo* survival remains incomplete, in part due to the technical challenges that face whole-genome bacterial screens *in vivo*. In this study, we leveraged a temporally resolved, high-coverage CRISPRi screen within an *ex vivo* platform that recapitulates the changing environment within the infected mouse lung. This novel strategy enabled us to identify context-dependent and conditionally essential Mtb genes and to demonstrate how their essentiality changed with the transition from a naïve to an immunologically experienced host. Because lung cell suspensions were derived from mice with established Mtb infection, the CRISPRi library encounters host cells conditioned by prior immune activation rather than a naïve intracellular environment. In consequence, the genetic dependencies identified here reflect bacterial requirements for survival and adaptation within experienced immune niches—conditions that may better approximate repeated exposure or reinfection. We would argue that this condition is the more frequent in highly endemic settings with high transmission, such as sub-Saharan Africa (11, 12).

Combining a comprehensive, inducible CRISPRi library with the deconstructed granuloma (DeGr) platform maintained the immunological complexity of the infected lung while permitting deep genetic coverage. This circumvents the major bottleneck in traditional *in vivo* screens, where aerosol or intranasal infection typically delivers too few colony-forming units (CFU) to achieve genome-scale saturation. The current approach also facilitates sampling across multiple infection time points and enables us to establish Mtb gene essentiality profiles at 2, 4, and 6 weeks post-infection, capturing the dynamic shifts in bacterial vulnerabilities as the immune environment matures. The deeper genetic coverage achieved by this approach facilitated replicate-correlation analysis (Fig. 2), which enabled discrimination between universally and environment-specific essentiality. Notably, the log_2_FC distributions revealed a substantially deeper tail at 2 weeks post-infection, indicating that many knockdown strains experience stronger depletion in early infection than in late infection. This early phase appears to be a period of heightened vulnerability in which the bacterium is dependent on a broader set of genes required to support active bacterial replication prior to transitioning into a state of lower replication in response to host immune-mediated stress.

At the early 2-week time point, the *embCAB* operon was a notable set of genes preferentially depleted. Our interpretation that arabinogalactan synthesis for cell wall components is of particular significance, because this is a period of rapid bacterial growth, is supported by earlier transcriptomic data indicating that Mtb metabolic activity peaks early in infection (17) and the induction of drug tolerance against INH and RIF peaks late (39). Both anti-TB drugs show preferential activity against actively growing Mtb, and this activity correlates inversely with the immune activation status of the host macrophage both *in vitro* and *in vivo* (39, 40). In contrast, genes involved in cholesterol catabolism and iron acquisition were required at all time points, consistent with Mtb’s reported dependence on host lipids and iron during chronic infection (5, 24, 34). Importantly, our findings further reveal that specific metabolic pathways—such as cholesterol side-chain degradation and the activity of the salicylate synthase *mbtI*—constitute potential chokepoints. This is consistent with the hypothesis that certain enzymatic reactions act as rate-limiting steps in critical metabolic pathways and may thus represent high-value drug targets (41).

While the DeGr-CRISPRi platform enables genome-wide analyses of bacterial genetic requirements within immune-conditioned host environments, there are some limitations. Tissue dissociation disrupts spatial lung architecture, the pooled format precludes assignment of bacterial dependencies to specific host cell subsets, and the model integrates survival in pre-infected rather than primary infection contexts. However, by preserving immune heterogeneity and enabling deep genetic coverage with temporal resolution, this approach provides a complementary framework for defining dynamic, context-dependent essentiality that is difficult to achieve using standard infection models.

In conclusion, our study offers a new route to the generation of a high-resolution genetic profile of Mtb gene essentiality at differing time points post-infection. By combining the precision of CRISPRi with an immune-informed *ex vivo* challenge model, we reveal the dynamic landscape of Mtb gene essentiality during infection and identify metabolic vulnerabilities that could inform the next generation of TB therapeutics. Moreover, this platform is broadly applicable and could be adapted to other pathogens or used to assess drug-gene interactions in the context of the host immune environment.

## MATERIALS AND METHODS

### Bacterial strains

*Mycobacterium tuberculosis* Erdman (American Type Culture Collection 35801) wild-type strain was utilized for intranasal infections and generation of heat-killed *M. tuberculosis* samples.

### Mice

C57BL/6 mice were purchased from the Jackson Laboratory. Mice were used at 6 to 10 weeks old. All mice were maintained in a specific pathogen-free, biosafety level 3 facility at Cornell University. Animal care was in compliance with the guidelines of the Association for Assessment and Accreditation of Laboratory Animal Care. All animal procedures were approved by the Institutional Animal Care and Use Committee of Cornell University.

### Generation of CRISPRi library

The *M. tuberculosis* CRISPRi Library RLC13 was designed to target all possible *M. tuberculosis* ORFs and non-coding RNAs with up to five sgRNAs and contains a total of 19,560 sgRNAs. RLC13 is a combination of five sub-libraries: RLC4, RLC5, RLC6, RLC7, and RLC8. Each individual sub-library (RLC4-RLC8) was designed to target all possible *M. tuberculosis* ORFs and non-coding RNAs with a single “strong” sgRNA (see definitions below).

To design each sub-library, we first extracted all possible sgRNA targeting sequences in the H37Rv *M. tuberculosis* genome (NC_018143.2) by identifying all 24 possible Sth1 dCas9 PAM sequences (13). We then extracted 22–24 nucleotide sgRNA targeting sequences upstream of each PAM. Only sgRNA targeting sequences in which the 5′ transcription initiating nucleotide was an “A” or “G” were kept for further processing. This list represented all possible Sth1 sgRNAs targeting the H37Rv genome. This list was further filtered to remove sgRNA targeting sequences containing an internal BsmBI restriction site and to ensure sgRNAs targeted the non-template strand of ORFs and non-coding RNAs. sgRNA targeting sequence overlap with an ORF or non-coding RNA was defined by the 3′ nucleotide of the sgRNA targeting sequence. sgRNAs targeting TnSeq-predicted essential genes (REF PMID: 28096490) (21) that did not demonstrate a median L2FC < –1 after passaging for 15 generations in the H37Rv competitive growth experiment (20) were discarded—this step prevented inclusion of inactive sgRNAs targeting essential genes.

For each gene, the five sgRNAs predicted to lead to the strongest inhibition based on PAM strength (13, 14) were selected, with the strongest guide assigned to RLC4, the next strongest to RLC5, and so on. Non-targeting control sgRNAs were also designed and included in the smaller libraries. To design non-targeting sgRNAs, the *Mtb* genome was scrambled, and sgRNAs were extracted according to the design principles listed above. This approach matches the GC content of targeting and non-targeting sgRNAs. Potential non-targeting sgRNAs were mapped back to NC_018143.2 using Bowtie (42). Only sgRNAs with at least two mismatches relative to the parental genome (and at least one mismatch in the sgRNA seed region, here defined as the PAM proximal 12 nucleotides) were selected as non-targeting sgRNAs for library construction.

In total, RLC13 contains 19,560 unique sgRNAs: 19,160 sgRNAs targeting *M. tuberculosis* genes and 400 non-targeting control sgRNAs. This represents 96.3% targeting coverage (3,972/4,125) of all *M. tuberculosis* ORFs and non-coding RNAs.

### CRISPRi library production

sgRNA targeting sequence oligonucleotides were designed to encode:

1. The sgRNA targeting sequence (21–24 nucleotides in length).
2. 5′ and 3′ BsmBI restriction sites with compatible sticky end DNA overhangs for sgRNA ligation into the CRISPRi plasmid backbone.
3. 5′ and 3′ primer binding sites for PCR amplification.

Oligonucleotides were synthesized by Agilent Technologies (SureGuide Custom CRISPR Guide Library #G7555B#100).

To generate each sublibrary (RLC4-RLC8), 250 μg of plRL2 (Addgene #163631) was digested with BsmBI (NEB #R0580) and gel-purified (Qiagen #28706). BsmBI-digested plRL2 was then further cleaned and concentrated by ethanol precipitation. Next, the pooled sgRNA oligonucleotide library was PCR-amplified using NEBNext High-Fidelity 2X PCR Master Mix (NEB #M0541L). Eight 50 μL PCR reactions were prepared for each sublibrary, with each reaction containing 25 μL of PCR master mix, 0.05 pmol of the oligonucleotide library, and a final concentration of 0.5 μM of the appropriate forward and reverse primers (RLC4: Fwd: 5′-CGCGTCGAGTAGGGT-3′ + Rev: 5′-GCCGTGTGAAGCTGG-3′; RLC5: Fwd: 5′-ACTGGTGCGTCGTCT-3′ + Rev: 5′-CGTCACGCAGGGTTC-3′; RLC6: Fwd: 5′-GGTCGAGCCGGAACT-3′ + Rev: 5′-AGCGAAACCGTGCGT-3′; RLC7: Fwd: 5′-AGTCGCGCCTACCAC-3′ + Rev: 5′-TGCGCCACCTCAGTC-3′; RLC8: Fwd: 5′-ACCGGTTTCCACGCA-3′ + Rev: 5′-TCCACCGTCGGCAAG-3′). PCR cycling conditions were as follows: 98°C for 30 s; 10 cycles of 98°C for 10 s, (RLC4: 65°C; RLC5: 64°C; RLC6-RLC8: 66°C) for 1:15, 65°C for 5:00. PCR amplicons were purified using the Qiagen MinElute PCR purification kit (Qiagen #28004). sgRNA targeting sequences were cloned into plRL2 by Golden Gate cloning (14). Each 20 μL Golden Gate reaction (six reactions for each sublibrary) contained 800 fmol of PCR amplicon, 80 fmol of BsmBI-digested plRL2, 20 mM DTT, 20 mM ATP, 1× FastDigest Buffer, 10 U of FastDigest Esp3I (Thermo Scientific #FD0454) and 1,000 units T4 DNA ligase (NEB #M0202M). Cycling conditions were as follows: 50 cycles of 37°C for 5 min and 16°C for 5 min, followed by 55°C for 1 h and a 4°C hold. Upon completion, 5 U of Esp3I was added per 20 μL reaction, and the mix was incubated at 37°C for 1 h. The Golden Gate reactions were terminated by heat killing at 80°C for 5 min. Following Golden Gate reactions, the products were purified and concentrated using a DNA Clean & Concentrator-25 kit (Zymo #D4034) and spot-dialyzed (Millipore #VSWP02500).

### *E. coli* library transformations

All CRISPRi sgRNA libraries were transformed into MegaX DH10B T1R Electrocompetent Cells (Invitrogen #C640003).

For each sublibrary, 1 μg of the dialyzed Golden Gate reaction was added to 200 μL of MegaX cells. The mixture was supplemented with 100 μL of ice-cold glycerol. A total of six transformations were performed for each sublibrary. For each transformation, 75 μL of the cells:DNA mix was transferred to a 0.1 cm electroporation cuvette (BioRad #1652089) and electroporated at 2,000 V, 200 ohms, and 25 μF. The transformations were recovered in a total of 10 mL SOC medium with shaking for 1.5 h at 37°C. Following recovery, bacteria were spread (650 μL per plate) on prewarmed LB Miller agar supplemented with kanamycin (50 μg/mL) in Corning Bioassay dishes (Sigma #CLS431111-16EA). The plates were incubated at 37°C for 18 h. Following outgrowth, transformants were harvested by scraping. The CRISPRi plasmid library was then isolated using a QIAGEN Plasmid Giga Kit (Qiagen #12191). Finally, the quality of the sgRNA library was confirmed by deep sequencing (see Library preparation for Illumina sequencing).

### CRISPRi library transformation

Validated CRISPRi libraries (see Genomic DNA extraction and library preparation for Illumina sequencing) were electroporated into mycobacteria as described (13).

Eight transformations per library were performed to generate *M. tuberculosis* H37Rv RLC4-RLC8 libraries. For each transformation, 800 ng of plasmid DNA was added to 200 μL electrocompetent cells (~4 × 10^9^ cells per transformation). The cells:DNA mix was transferred to a 2 mm electroporation cuvette (Bio-Rad #1652082) and electroporated at 2,500 kV, 700 ohms, and 25 μF. Each transformation was recovered in 5 mL 7H9 media supplemented with OADC, glycerol, and Tween 80 for 16–24 h. The recovered cells were harvested at 4,000 rpm for 10 min, resuspended in 1 mL remaining media per transformation, and plated on 7H10 agar supplemented with OADC, glycerol, Tween 80, and kanamycin in Corning Bioassay dishes (Sigma #CLS431111-16EA). Transformation efficiency was estimated from library titering and indicated ~70–120× average sgRNA coverage of each library was achieved in *M. tuberculosis*.

After 21 days of outgrowth on plates, transformants were scraped and pooled. Scraped cells were homogenized by two dissociation cycles on a gentleMACS Octo Dissociator (Miltenyi Biotec #130095937) using the RNA_01 program and gentleMACS M tubes (Miltenyi Biotec #130093236). Each library was further declumped by passaging 10 individual *M. tuberculosis* library aliquots in 10 mL of 7H9 supplemented with OADC, glycerol, Tween 80, and kanamycin in T-25 flasks (Falcon #08-772-1F) for 15 generations. Final *M. tuberculosis* library stocks were obtained after pooling the cultures and passing them through a 10 μm cell strainer (Pluriselect #SKU 43-50010-03). Genomic DNA was extracted from the final *M. tuberculosis* RLC4-RLC8 library stocks, and library quality was validated by deep sequencing (see Genomic DNA extraction and library preparation for Illumina sequencing).

To generate RLC13, 1 mL aliquots of the final homogenized RLC4-RLC8 libraries were thawed. Each aliquot was inoculated into 24 mL 7H9 media supplemented with kanamycin in a T-75 flask (starting OD_600_ ~0.02, which represents ~30,000× library coverage). The cultures were expanded to OD_600_ ~2, passed through a 10 μm cell strainer (PluriSelect #43-50010-03) to obtain a single-cell suspension, and equal cell numbers from each library were pooled. The OD_600_ of the mixture was adjusted to 1.0, and aliquots were frozen at –80°C. Genomic DNA was extracted from the pooled library, and library quality was validated by deep sequencing (see Genomic DNA extraction and library preparation for Illumina sequencing).

### Vaccination of mice

Wild-type Erdman *M. tuberculosis* cultures were harvested by centrifugation and resuspended in sterile phosphate-buffered saline (PBS) to an OD_600_ of 0.6, corresponding to approximately 1 × 10^7^ CFU/mL. Suspensions were aliquoted into 1 mL screw-cap tubes and heat-inactivated at 90°C for 30 min using a dry heat block. Complete inactivation was verified for each batch by plating 100 µL aliquots from duplicate tubes/batch and incubating the plates for 30 days at 37°C, with the absence of growth indicating successful inactivation. Mice were vaccinated intraperitoneally with heat-killed *M. tuberculosis* suspended in PBS at a dose of 1 × 10^6^ bacteria in 100 µL/mouse. Intranasal infection followed 4 weeks post-vaccination.

### Generation of deconstructed granuloma (DeGr) cell suspensions

Mice were infected intranasally with 1,000 CFU of the Erdman strain of *Mtb* and then sacrificed at desired time points post-infection (2, 4, or 6 w.p.i.). Lung lobes were isolated, placed in C-tubes containing dissociation buffer (5% FBS and 250 U/mL collagenase IV in PBS), and homogenized using a Miltenyi GentleMACS tissue homogenizer. Homogenates were treated with 1 mL ACK buffer for 5 min to lyse RBCs. Samples were then resuspended in wash buffer (5% FBS + 50 µg/mL gentamycin in PBS) and taken through a series of straining (70 and 40 μM cell strainers) and washing steps in wash buffer. Gentamicin was used to eliminate extracellular contaminating bacteria and should not impact those bacteria which reside within host cells given gentamicin’s inability to penetrate mammalian cells. The final wash was performed in antibiotic-free 5% FBS in PBS to remove any gentamicin prior to *ex vivo* challenge. Cells were then resuspended in complete DMEM (see “Reagent recipes” section). Lung cells were then plated in T-75 flasks (~45 million cells/flask; typically four flasks per harvest) to generate DeGr single-cell suspensions. DeGr cells were left to rest for at least 3 h prior to *ex vivo* challenge with *Mtb* CRISPRi library. If a harvest did not have enough cells for four flasks, then the remaining three flasks were pooled together as one technical replicate. Therefore, some rounds had one output final sample (pooling of two flasks), while some had two outputs (pooling of three flasks). “Round” corresponds to an independent experiment, whereas “output” corresponds to a technical replicate (pooling of three or four flasks) within an experiment.

### Reagent recipes

#### Complete DMEM

10% FBS

1 mM sodium pyruvate

2 mM L-glutamine

20 µg/mL Amphotericin B (to prevent fungal contamination)

DMEM

#### Basal uptake buffer

25 mM dextrose

0.5% bovine serum albumin

0.1% gelatin

1 mM CaCl_2_

0.5 mM MgCl_2_

PBS

### Set-up of CRISPRi genetic screens

The CRISPRi library was cultured in 7H9 OADC with 25 µg/mL kanamycin for 4 days. The library was grown to mid-log phase in a roller bottle to achieve a homogenous culture. Twenty-four hours prior to *ex vivo* challenge of lung cells, anhydrous tetracycline (ATc) was added to the library (at a final concentration of 100 ng/mL) to induce expression of dCas9 and sgRNA. At time of *ex vivo* challenge, the CRISPRi library was centrifuged, resuspended in Basal Uptake Buffer (BUB), passed through a tuberculin syringe 10 times, and resuspended in complete DMEM media. DeGr lung cells were then *ex vivo* challenged with the bacterial suspension at an MOI of 0.05 (~2.25 million bacterial cells/flask). Genome coverage of the library was >100-fold for each flask. ATc was maintained at a final concentration of 100 ng/mL, and flasks were incubated in a CO_2_ incubator at 37°C. While we cannot unequivocally state that all bacteria are intracellular, the extended incubation period, the excess of phagocytic cells, and the fact that the selected mutations have intracellular phenotypes all indicate that the selection pressure is most acute for intracellular bacilli.

### Bacterial genomic DNA isolation

At the time of *ex vivo* challenge (T0), 10 mL of the input library from broth culture was pelleted in GTC (see “Reagent recipes” section) and frozen at −80°C. DeGr cells were monitored via light microscope and harvested between 6 and 8 days post *ex vivo* challenge. Briefly, the culture supernatants were removed and placed in labeled 50 mL conical tubes. Fifteen milliliters of cold PBS were added to T-75 flasks, which were then placed at 4°C. After 10 min at 4°C, the adherent cells were removed using a cell scraper. These lysates were then combined with their respective supernatants in the labeled 50 mL conical tubes. All cells were pelleted at 1,000 rpm for 10 min. The pellets were resuspended in 25 mL of GTC buffer to chemically lyse the murine cells. These lysates were vortexed for 2 min and then syringed five times using a 21 ga needle and 20 mL syringe to break up nuclear DNA. Samples were centrifuged at 3,500 × *g* for 40 min to pellet bacteria. The pellets were resuspended in 2 mL of GTC and placed at −80°C for future genomic DNA (gDNA) isolation.

Input and output samples in GTC were slowly thawed at room temperature on the day of gDNA isolation. Thawed samples were centrifuged at 15,000 rpm for 5 min and washed once in 2 mL of PBS + 0.05% Tween 80 (PBST-80) to remove residual GTC. Samples in PBST-80 were centrifuged and washed in 1 mL of GTE buffer (see “Reagent recipes” section below). Pellets were resuspended in 450 µL of GTE and 50 µL of lysozyme (see “Reagent recipes” section) and incubated overnight at 37°C.

The following day, CTAB solution (see “Reagent recipes” section) was preheated to 65°C. One hundred microliters of SDS solution (10% sodium dodecyl sulfate in water) and 50 µL of proteinase K (10 mg/mL in GTE) were added to the lysozyme samples, which were then incubated at 55°C for 30 min. Two hundred microliters of 5 M NaCl and 160 µL of preheated CTAB were then added, and the samples were incubated at 65°C for 10 min. Next, 900 µL of 24:1 chloroform:isoamyl solution was added, and the tubes were inverted gently prior to centrifugation at 15,000 × *g* for 10 min. Approximately 800 µL of the aqueous layer was transferred to a new tube, and samples were decontaminated for transfer to the BSL-2 lab for protocol completion.

DNA was precipitated by adding 560 µL of isopropanol and 2 µL of GlycoBlue co-precipitate for pellet visualization, followed by a 10-minute incubation at room temperature. Precipitated samples were centrifuged at 15,000 × *g* for 10 min, and the pellets were washed in 1 mL of 70% ethanol. The washed pellets were dried for 15–20 min to remove residual ethanol. Finally, the DNA was dissolved in 20 µL of Qiagen Elution Buffer.

### Library preparation for Illumina sequencing

Genomic DNA concentration was quantified using the DeNovix dsDNA high-sensitivity assay (KIT-DSDNA-HIGH-2; DS-11 Series Spectrophotometer/Fluorometer). Next, the sgRNA-encoding region was amplified from 30 to 75 ng of genomic DNA using NEBNext Ultra II Q5 Master Mix (NEB #M0544L). PCR cycling conditions were as follows: 98°C for 45 s; 23–25 cycles of 98°C for 10 s, 64°C for 30 s, 65°C for 20 s; 65°C for 5 min. Samples were single-indexed or dual-indexed. For single-indexed samples, each PCR reaction contained a pool of forward primers (0.5 μM final concentration) and a unique indexed reverse primer (0.5 μM). For dual-indexed samples, each PCR reaction contained a unique indexed forward and reverse primer (0.5 μM each). Forward primers contain a P5 flow cell attachment sequence, a standard Read1 Illumina sequencing primer binding site, custom stagger sequences to ensure base diversity during Illumina sequencing, and a unique barcode in the case of dual-indexed samples. Reverse primers contain a P7 flow cell attachment sequence, a standard Read2 Illumina sequencing primer binding site, and unique barcodes to allow for sample pooling during deep sequencing.

Following PCR amplification, each ~230 bp amplicon was purified using sparQ PureMag Beads (Quantabio #95196-060) with single-sided size selection (1.2×) or double-sided size selection (first 0.75×, then an additional 0.12× for a final 0.87×). For single-sided bead-purified amplicons, samples were further purified on a Pippin HT 2% agarose gel cassette (target range 180–250; Sage Science #HTC2010) to remove primer carryover and genomic DNA before quantification and quality control. For double-sided selection, eluted samples were immediately quantified and quality controlled. Eluted, size-selected amplicons were quantified with a Qubit 2.0 Fluorometer (Invitrogen). Amplicon size and purity were quality controlled by visualization on an Agilent 2100 Bioanalyzer (high sensitivity chip; Agilent Technologies #5067-4626). Next, individual PCR amplicons were multiplexed into 10 nM pools and sequenced on an Illumina sequencer according to the manufacturer’s instructions (2.5–5% PhiX spike-in; PhiX Sequencing Control v3; Illumina #FC-110-3001). Samples were run on the Illumina NextSeq 500 platform (single-read 1 × 85 cycles and six i7 index cycles). Samples were sequenced to achieve a target sgRNA median count depth of approximately >200 per sample.

### Data processing and hit-calling

Sequencing data were processed in the manner described by Bosch et al. (2021) (20). Read counts were normalized for sequencing depth, and a limit of detection (LOD) cutoff was set at 20 counts in the input conditions. Only those sgRNAs that made the LOD cutoff (i.e., read counts >20) were analyzed further. sgRNA counts in the input conditions were compared to the corresponding experimental conditions using MAGeCK (version 0.5.9.2). Log2 fold change (L2FC) was calculated using the “alphamedian” approach specified with the “gene-lfc-method” parameter, which estimates the gene-level L2FC as the median of sgRNAs that are ranked above the default cutoff in the Robust Rank Analysis used by MAGeCK. Counts were normalized based on negative control guides using the “–normalization control” parameter, and a null distribution was constructed using the negative control guides by specifying the “--control-sgrna” parameter.

Genes were considered to be a hit if they had a false discovery rate <0.01, an |L2FC| > 1, and at least 50% of their sgRNAs passing the significance threshold (*P* < 0.10) in MAGeCk’s initial sgRNA-level analysis.

### Generation of CRISPRi knockdown strains for validation

For individual knockdown strains, two sgRNAs were designed for each gene. Oligos with the targeting sequence flanked by BsmBI overhangs were ordered from IDT. Four microliters of each oligo were added to 42 µL of NEB Buffer 3.1, and the oligos were annealed with the following thermocycler conditions: 95°C for 2 min, 0.1°C/sec to 25°C. The pIRL2 CRISPRi plasmid was amplified and digested with BsmBI, followed by gel purification of the digested backbone. 0.5 µL of annealed oligos and ~9 ng of the digested vector were ligated using NEB T4 ligase. The ligation product was transformed into TOP10 *E. coli*, and selection was performed on LB + 50 µg/mL kanamycin. Plasmid DNA was isolated from selected colonies using a Qiagen Miniprep kit. The sequencing primer (5′-TTCCTGTGAAGAGCCATTGATAATG-3′) was used for confirmation of sgRNA insertion.

Validated constructs were then transformed into electrocompetent Erdman Mtb, and clones were selected following growth on 7H10 plates + OADC + 25 µg/mL kanamycin.

### Assessment of sgRNA efficiency for individual knockdown strains

CRISPRi strains were grown in 100 mL 7H9 OADC + Kan-25 in roller bottles for 2 days starting at OD 0.02. ATc was added at 200 ng/mL when the cultures reached ~OD 0.2–0.4 to induce gene knockdown. At 4, 8, 24, and 72 h post-induction, 10 mL of culture was centrifuged at 3,000 rpm for 10 min, and pellets were resuspended in 2 mL of GTC before being placed at −80°C.

GTC samples were thawed at the time of RNA isolation procedure. Samples were first washed in 2 mL of PBST-80 to remove residual GTC. Pellets were resuspended in 250 µL of 5 mg/mL lysozyme in water and incubated at room temperature for 15 min. 0.75 mL of warm TRIzol (prewarmed to 65°C) was added to each sample. Samples were transferred to 2 mL tubes containing 0.1 mm glass beads and placed on a bead beater for 2 min. Lysed samples were rested for 2 min prior to the addition of 200 µL of chloroform. Tubes were gently inverted several times and centrifuged at 12,000 rpm for 15 min to separate phases. Approximately 700 µL of the aqueous phase was transferred to tubes containing 500 µL of pure ethanol. A Qiagen RNeasy kit was used to complete the RNA isolation procedure, and RNA was eluted into 32 µL of NCFW. The TURBO DNase procedure was used for removal of genomic DNA.

One hundred nanograms of RNA were converted to cDNA using the iSCRIPT cDNA synthesis kit (Bio-Rad). RT-qPCR plates were set up using SYBR Green master mix from Bio-Rad (2 µL of cDNA template, 2.5 µL of 10 µM FWD primer, 2.5 µL of 10 µM REV primer, 12.5 µL of SYBR master mix, and 5.5 µL of NCFW). *sigA* was used as an endogenous control. ΔΔCT values were calculated relative to *sigA* endogenous and no ATc (uninduced) controls. The sgRNA that caused a larger reduction in log2fc was used for *in vivo* validation experiments.

### *In vivo* mouse validation experiments

Mice were infected intranasally with 1,000 CFU of the individual knockdown strains. Five days post-infection, mice were fed doxycycline chow (Research Diets C11300-2000i) to induce knockdown. Control mice were continued to be fed normal mouse chow. Treated and control mice were euthanized at 2 and 3 w.p.i. Whole lungs were removed and manually digested using dissection scissors followed by homogenization in bullet blender tubes containing 1 mL of PBS + 0.05% Tween 80. Lung homogenates were then serially diluted and plated on 7H10 plates for CFU enumeration.

## SUPPLEMENTAL MATERIAL

File S1 mbio.00610-26-s0001.xlsx Output table of the genome-wide CRISPRi screens.

File S2 mbio.00610-26-s0002.xlsx Replicate-correlation analysis file containing pairwise comparison data for all time points.

File S3 mbio.00610-26-s0003.xlsx Gene lists used for the Venn diagram analysis.

File S4 mbio.00610-26-s0004.xlsx Genetic screening results for key biological pathways highlighted in the study.

File S5 mbio.00610-26-s0005.xlsx Total lung CFU measurements from all *in vivo* validation experiments.

Legends mbio.00610-26-s0006.docx Descriptions of supplemental files.

ASM does not own the copyrights to Supplemental Material that may be linked to, or accessed through, an article. The authors have granted ASM a non-exclusive, world-wide license to publish the Supplemental Material files. Please contact the corresponding author directly for reuse.

## References

[1] Bagcchi S. 2023. WHO's global tuberculosis report 2022. Lancet Microbe 4:e20. doi:10.1016/S2666-5247(22)00359-7 36521512

[2] Behr MA, Kaufmann E, Duffin J, Edelstein PH, Ramakrishnan L. 2021. Latent tuberculosis: two centuries of confusion. Am J Respir Crit Care Med 204:142–148. doi:10.1164/rccm.202011-4239PP 33761302 PMC8650795

[3] Houben R, Dodd PJ. 2016. The global burden of latent tuberculosis infection: a re-estimation using mathematical modelling. PLoS Med 13:e1002152. doi:10.1371/journal.pmed.1002152 27780211 PMC5079585

[4] Farhana A, Guidry L, Srivastava A, Singh A, Hondalus MK, Steyn AJC. 2010. Reductive stress in microbes: implications for understanding Mycobacterium tuberculosis disease and persistence. Adv Microb Physiol 57:43–117. doi:10.1016/B978-0-12-381045-8.00002-3 21078441

[5] Russell DG, Huang L, VanderVen BC. 2019. Immunometabolism at the interface between macrophages and pathogens. Nat Rev Immunol 19:291–304. doi:10.1038/s41577-019-0124-9 30679807 PMC7032560

[6] Sassetti CM, Rubin EJ. 2003. Genetic requirements for mycobacterial survival during infection. Proc Natl Acad Sci USA 100:12989–12994. doi:10.1073/pnas.2134250100 14569030 PMC240732

[7] Bellerose MM, Proulx MK, Smith CM, Baker RE, Ioerger TR, Sassetti CM. 2020. Distinct bacterial pathways influence the efficacy of antibiotics against Mycobacterium tuberculosis. mSystems 5:e00396-20. doi:10.1128/mSystems.00396-20 32753506 PMC7406225

[8] Block AM, Wiegert PC, Namugenyi SB, Tischler AD. 2024. Transposon sequencing reveals metabolic pathways essential for Mycobacterium tuberculosis infection. PLoS Pathog 20:e1011663. doi:10.1371/journal.ppat.1011663 38498580 PMC10977890

[9] James KS, Jain N, Witzl K, Cicchetti N, Fortune SM, Ioerger TR, Martinot AJ, Carey AF. 2025. TnSeq identifies genetic requirements of Mycobacterium tuberculosis for survival under vaccine-induced immunity. NPJ Vaccines 10:103. doi:10.1038/s41541-025-01150-9 40404665 PMC12098976

[10] Zhang YJ, Reddy MC, Ioerger TR, Rothchild AC, Dartois V, Schuster BM, Trauner A, Wallis D, Galaviz S, Huttenhower C, Sacchettini JC, Behar SM, Rubin EJ. 2013. Tryptophan biosynthesis protects mycobacteria from CD4 T-cell-mediated killing. Cell 155:1296–1308. doi:10.1016/j.cell.2013.10.045 24315099 PMC3902092

[11] Dinkele R, Gessner S, Patterson B, McKerry A, Hoosen Z, Vazi A, Seldon R, Koch A, Warner DF, Wood R. 2024. Persistent Mycobacterium tuberculosis bioaerosol release in a tuberculosis-endemic setting. iScience 27:110731. doi:10.1016/j.isci.2024.110731 39310776 PMC11414687

[12] Warner DF, Barczak AK, Gutierrez MG, Mizrahi V. 2025. Mycobacterium tuberculosis biology, pathogenicity and interaction with the host. Nat Rev Microbiol 23:788–804. doi:10.1038/s41579-025-01201-x 40588584 PMC7618155

[13] Rock JM, Hopkins FF, Chavez A, Diallo M, Chase MR, Gerrick ER, Pritchard JR, Church GM, Rubin EJ, Sassetti CM, Schnappinger D, Fortune SM. 2017. Programmable transcriptional repression in mycobacteria using an orthogonal CRISPR interference platform. Nat Microbiol 2:16274. doi:10.1038/nmicrobiol.2016.274 28165460 PMC5302332

[14] Wong AI, Rock JM. 2021. CRISPR interference (CRISPRi) for targeted gene silencing in mycobacteria. Methods Mol Biol 2314:343–364. doi:10.1007/978-1-0716-1460-0_16 34235662

[15] Huang L, Kushner NL, Theriault ME, Pisu D, Tan S, McNamara CW, Petrassi HM, Russell DG, Brown AC. 2018. The deconstructed granuloma: a complex high-throughput drug screening platform for the discovery of host-directed therapeutics against tuberculosis. Front Cell Infect Microbiol 8:275. doi:10.3389/fcimb.2018.00275 30155446 PMC6102409

[16] Huang L, Nazarova EV, Tan S, Liu Y, Russell DG. 2018. Growth of Mycobacterium tuberculosis in vivo segregates with host macrophage metabolism and ontogeny. J Exp Med 215:1135–1152. doi:10.1084/jem.20172020 29500179 PMC5881470

[17] Pisu D, Huang L, Grenier JK, Russell DG. 2020. Dual RNA-Seq of Mtb-infected macrophages in vivo reveals ontologically distinct host-pathogen interactions. Cell Rep 30:335–350. doi:10.1016/j.celrep.2019.12.033 31940480 PMC7032562

[18] Pisu D, Huang L, Narang V, Theriault M, Lê-Bury G, Lee B, Lakudzala AE, Mzinza DT, Mhango DV, Mitini-Nkhoma SC, Jambo KC, Singhal A, Mwandumba HC, Russell DG. 2021. Single cell analysis of M. tuberculosis phenotype and macrophage lineages in the infected lung. J Exp Med 218:e20210615. doi:10.1084/jem.20210615 34292313 PMC8302446

[19] Pisu D, Johnston L, Mattila JT, Russell DG. 2024. The frequency of CD38^+^ alveolar macrophages correlates with early control of M. tuberculosis in the murine lung. Nat Commun 15:8522. doi:10.1038/s41467-024-52846-w 39358361 PMC11447019

[20] Bosch B, DeJesus MA, Poulton NC, Zhang W, Engelhart CA, Zaveri A, Lavalette S, Ruecker N, Trujillo C, Wallach JB, Li S, Ehrt S, Chait BT, Schnappinger D, Rock JM. 2021. Genome-wide gene expression tuning reveals diverse vulnerabilities of M. tuberculosis. Cell 184:4579–4592. doi:10.1016/j.cell.2021.06.033 34297925 PMC8382161

[21] DeJesus MA, Gerrick ER, Xu W, Park SW, Long JE, Boutte CC, Rubin EJ, Schnappinger D, Ehrt S, Fortune SM, Sassetti CM, Ioerger TR. 2017. Comprehensive essentiality analysis of the Mycobacterium tuberculosis genome via saturating transposon mutagenesis. mBio 8:e02133-16. doi:10.1128/mBio.02133-16 28096490 PMC5241402

[22] Griffin JE, Gawronski JD, Dejesus MA, Ioerger TR, Akerley BJ, Sassetti CM. 2011. High-resolution phenotypic profiling defines genes essential for mycobacterial growth and cholesterol catabolism. PLoS Pathog 7:e1002251. doi:10.1371/journal.ppat.1002251 21980284 PMC3182942

[23] Gill WP, Harik NS, Whiddon MR, Liao RP, Mittler JE, Sherman DR. 2009. A replication clock for Mycobacterium tuberculosis. Nat Med 15:211–214. doi:10.1038/nm.1915 19182798 PMC2779834

[24] Pandey AK, Sassetti CM. 2008. Mycobacterial persistence requires the utilization of host cholesterol. Proc Natl Acad Sci USA 105:4376–4380. doi:10.1073/pnas.0711159105 18334639 PMC2393810

[25] Miner MD, Chang JC, Pandey AK, Sassetti CM, Sherman DR. 2009. Role of cholesterol in Mycobacterium tuberculosis infection. Indian J Exp Biol 47:407–411. 19634704

[26] Muñoz-Elías EJ, Upton AM, Cherian J, McKinney JD. 2006. Role of the methylcitrate cycle in Mycobacterium tuberculosis metabolism, intracellular growth, and virulence. Mol Microbiol 60:1109–1122. doi:10.1111/j.1365-2958.2006.05155.x 16689789

[27] Lee W, VanderVen BC, Fahey RJ, Russell DG. 2013. Intracellular Mycobacterium tuberculosis exploits host-derived fatty acids to limit metabolic stress. J Biol Chem 288:6788–6800. doi:10.1074/jbc.M112.445056 23306194 PMC3591590

[28] McKinney JD, zu Bentrup KH, Muñoz-Elías EJ, Miczak A, Chen B, Chan W-T, Swenson D, Sacchettini JC, Jacobs WR Jr, Russell DG. 2000. Persistence of Mycobacterium tuberculosis in macrophages and mice requires the glyoxylate shunt enzyme isocitrate lyase. Nature 406:735–738. doi:10.1038/35021074 10963599

[29] VanderVen BC, Fahey RJ, Lee W, Liu Y, Abramovitch RB, Memmott C, Crowe AM, Eltis LD, Perola E, Deininger DD, Wang T, Locher CP, Russell DG. 2015. Novel inhibitors of cholesterol degradation in Mycobacterium tuberculosis reveal how the bacterium’s metabolism is constrained by the intracellular environment. PLoS Pathog 11:e1004679. doi:10.1371/journal.ppat.1004679 25675247 PMC4335503

[30] Wilburn KM, Fieweger RA, VanderVen BC. 2018. Cholesterol and fatty acids grease the wheels of Mycobacterium tuberculosis pathogenesis. Pathog Dis 76:fty021. doi:10.1093/femspd/fty021 29718271 PMC6251666

[31] Rodriguez GM, Smith I. 2003. Mechanisms of iron regulation in mycobacteria: role in physiology and virulence. Mol Microbiol 47:1485–1494. doi:10.1046/j.1365-2958.2003.03384.x 12622807

[32] Boelaert JR, Vandecasteele SJ, Appelberg R, Gordeuk VR. 2007. The effect of the host’s iron status on tuberculosis. J Infect Dis 195:1745–1753. doi:10.1086/518040 17492589

[33] Theriault ME, Pisu D, Wilburn KM, Lê-Bury G, MacNamara CW, Michael Petrassi H, Love M, Rock JM, VanderVen BC, Russell DG. 2022. Iron limitation in M. tuberculosis has broad impact on central carbon metabolism. Commun Biol 5:685. doi:10.1038/s42003-022-03650-z 35810253 PMC9271047

[34] Siegrist MS, Unnikrishnan M, McConnell MJ, Borowsky M, Cheng T-Y, Siddiqi N, Fortune SM, Moody DB, Rubin EJ. 2009. Mycobacterial Esx-3 is required for mycobactin-mediated iron acquisition. Proc Natl Acad Sci USA 106:18792–18797. doi:10.1073/pnas.0900589106 19846780 PMC2774023

[35] Serafini A, Pisu D, Palù G, Rodriguez GM, Manganelli R. 2013. The ESX-3 secretion system is necessary for iron and zinc homeostasis in Mycobacterium tuberculosis. PLoS One 8:e78351. doi:10.1371/journal.pone.0078351 24155985 PMC3796483

[36] Zwahlen J, Kolappan S, Zhou R, Kisker C, Tonge PJ. 2007. Structure and mechanism of MbtI, the salicylate synthase from Mycobacterium tuberculosis. Biochemistry 46:954–964. doi:10.1021/bi060852x 17240979

[37] Harrison AJ, Yu M, Gårdenborg T, Middleditch M, Ramsay RJ, Baker EN, Lott JS. 2006. The structure of MbtI from Mycobacterium tuberculosis, the first enzyme in the biosynthesis of the siderophore mycobactin, reveals it to be a salicylate synthase. J Bacteriol 188:6081–6091. doi:10.1128/JB.00338-06 16923875 PMC1595383

[38] Rodriguez GM, Voskuil MI, Gold B, Schoolnik GK, Smith I ideR. 2002. ideR, An essential gene in Mycobacterium tuberculosis: role of IdeR in iron-dependent gene expression, iron metabolism, and oxidative stress response. Infect Immun 70:3371–3381. doi:10.1128/IAI.70.7.3371-3381.2002 12065475 PMC128082

[39] Liu Y, Tan S, Huang L, Abramovitch RB, Rohde KH, Zimmerman MD, Chen C, Dartois V, VanderVen BC, Russell DG. 2016. Immune activation of the host cell induces drug tolerance in Mycobacterium tuberculosis both in vitro and in vivo. J Exp Med 213:809–825. doi:10.1084/jem.20151248 27114608 PMC4854729

[40] Kirubakar G, Johnston L, Nae Rin Lee B, Russell DG, Simwela NV. 2025. Incorporation of macrophage immune stresses into an intracellular assay of drug tolerance in Mycobacterium tuberculosis. Antimicrob Agents Chemother 69:e0079525. doi:10.1128/aac.00795-25 40934365 PMC12486812

[41] Murima P, McKinney JD, Pethe K. 2014. Targeting bacterial central metabolism for drug development. Chem Biol 21:1423–1432. doi:10.1016/j.chembiol.2014.08.020 25442374

[42] Langmead B, Trapnell C, Pop M, Salzberg SL. 2009. Ultrafast and memory-efficient alignment of short DNA sequences to the human genome. Genome Biol 10:R25. doi:10.1186/gb-2009-10-3-r25 19261174 PMC2690996

