# Erratum for Theriault et al., “Utilization of a CRISPRi-based *ex vivo* challenge model to reveal temporally dependent gene essentiality in intracellular *Mycobacterium tuberculosis*”

**DOI:** 10.1128/mbio.01517-26

**Published:** 2026-06-26

**Authors:** Monique E. Theriault, Andrew I. Wong, Michael A. DeJesus, Davide Pisu, Bom Nae Rin Lee, Greana Kirubakar, Shuqi Li, Joshua B. Wallach, Dirk Schnappinger, Gabrielle Lê-Bury, David G. Russell, Jeremy M. Rock

## ERRATUM

Volume 17, no. 5, e00610-26, 2025, https://doi.org/10.1128/mbio.00610-26. Byline: Greana Kirubakar’s name should appear as shown in this Erratum.

