# Supplementary material for: Utilization of a CRISPRi-based ex vivo challenge model to reveal temporally dependent gene essentiality in intracellular Mycobacterium tuberculosis
Source: mBio. 2026 Apr 20;17(5):e00610-26. doi: 10.1128/mbio.00610-26 (PMC13170276; doi:10.1128/mbio.00610-26)
Supplement: Legends — Descriptions of supplemental files. [file mbio.00610-26-s0006.docx]

**Description of Supplementary files**

**Supplementary File 1.** Output table of the genome-wide CRISPRi screens across all timepoints and biological replicates, including log₂ fold-change (log₂FC) values and associated p-values for every sgRNA.

**Supplementary File 2.** Replicate-correlation analysis file containing pairwise comparison data for all timepoints, with each replicate–replicate correlation presented in a separate tab.

**Supplementary File 3.** Gene lists used for the Venn diagram analysis, including duplicate-filtered essential gene sets and overlap outputs for each infection timepoint.

**Supplementary File 4.** Genetic screening results for key biological pathways highlighted in the study, including *embCAB* cell-wall biosynthesis, cholesterol catabolism, and iron-homeostasis modules.

**Supplementary File 5**. Total lung CFU measurements from all *in vivo* validation experiments corresponding to CRISPRi knockdown strains.
